# Long Chain Fatty Acids and Virulence Repression in Intestinal Bacterial Pathogens

**DOI:** 10.3389/fcimb.2022.928503

**Published:** 2022-06-17

**Authors:** Mary K. Mitchell, Melissa Ellermann

**Affiliations:** Department of Biological Sciences, University of South Carolina, Columbia, SC, United States

**Keywords:** long chain fatty acids, lipid signaling, enteric infection, bacterial virulence, bacterial pathogenesis, two-component systems

## Abstract

When bacterial pathogens enter the gut, they encounter a complex milieu of signaling molecules and metabolites produced by host and microbial cells or derived from external sources such as the diet. This metabolomic landscape varies throughout the gut, thus establishing a biogeographical gradient of signals that may be sensed by pathogens and resident bacteria alike. Enteric bacterial pathogens have evolved elaborate mechanisms to appropriately regulate their virulence programs, which involves sensing and responding to many of these gut metabolites to facilitate successful gut colonization. Long chain fatty acids (LCFAs) represent major constituents of the gut metabolome that can impact bacterial functions. LCFAs serve as important nutrient sources for all cellular organisms and can function as signaling molecules that regulate bacterial metabolism, physiology, and behaviors. Moreover, in several enteric pathogens, including *Salmonella enterica, Listeria monocytogenes, Vibrio cholerae*, and enterohemorrhagic *Escherichia coli*, LCFA sensing results in the transcriptional repression of virulence through two general mechanisms. First, some LCFAs function as allosteric inhibitors that decrease the DNA binding affinities of transcriptional activators of virulence genes. Second, some LCFAs also modulate the activation of histidine kinase receptors, which alters downstream intracellular signaling networks to repress virulence. This mini-review will summarize recent studies that have investigated the molecular mechanisms by which different LCFA derivatives modulate the virulence of enteric pathogens, while also highlighting important gaps in the field regarding the roles of LCFAs as determinants of infection and disease.

## Introduction

The mammalian gastrointestinal (GI) tract is a dynamic metabolic environment that harbors a community of microbes known as the gut microbiota. The factors that modulate microbial niche availability throughout the gut varies temporally and geospatially due to diverse host, environmental, and microbial factors ranging from diet to host physiology and inflammation. Enteric bacterial pathogens must navigate these changing metabolic landscapes and compete with endogenous microbes to successfully colonize the gut. To accomplish this feat, pathogens deploy a suite of virulence factors including pili, toxins, and type III secretion systems to establish a replicative niche, which disrupts homeostatic intestinal functions and can result in disease. Despite their necessity for pathogen colonization, many virulence factors are energetically costly to produce (Sturm et al., 2011; Diard et al., 2013; Vasanthakrishnan et al., 2015; Davis, 2020; Hockenberry et al., 2021). Consequently, the expression of virulence genes is tightly regulated by transcription factors and complex signaling networks that link pathogen sensing of the environment to optimal activation of their virulence programs (Pacheco and Sperandio, 2015; Nisco et al., 2018).

Nutrients such as sugars, amino acids, and fatty acids often act as signaling molecules that regulate virulence in enteric pathogens. Throughout the intestines, the concentrations of specific nutrients derived from the diet or from host or microbial metabolism vary longitudinally along the tract and radially from the lumen to the mucosal-epithelial interface. This establishes a geospatial map of signals that may be sensed by invading pathogens and contribute to their virulence regulation. This mini-review will focus on a class of lipids known as long chain fatty acids and the mechanisms by which they regulate virulence in enteric pathogens.

## Long Chain Fatty Acids

Long chain fatty acids (LCFAs) are non-esterified fatty acids 12-20 carbons in length with varying degrees of saturation. Because of their dual roles as nutrients and signaling molecules, LCFAs modulate bacterial growth and functions through various mechanisms. LCFAs serve as essential building blocks for membrane biosynthesis and can be catabolized for energy *via* beta-oxidation by some bacterial taxa (Fujita et al., 2007). Additionally, LCFAs directly act as signaling molecules that regulate diverse functions in bacteria – including virulence (Prasun et al., 2020). Some bacterial taxa modify LCFA substrates to produce bioactive metabolites such as diffusible signal factors, *cis*-2-unsaturated LCFAs that function as quorum sensing signals (Dow, 2017). Finally, some LCFAs exhibit antimicrobial properties against certain bacterial taxa (Prasun et al., 2020). Thus, LCFAs impact bacterial growth, metabolism, physiology, and behaviors in complex ways including through their roles as environmental signals.

Intestinal LCFAs are likely derived from dietary sources, host cells, and microbial metabolites. The most abundant LCFAs within the lumen include unsaturated fatty acids such as oleic acid and linoleic acid, and saturated fatty acids such as stearic acid and palmitic acid (Batta et al., 2002; Metzler-Zebeli et al., 2021). As with other nutrients, the relative abundances of LCFAs vary throughout the GI tract because of numerous factors that likely include host diet, bile acid secretions, and microbial metabolism (Metzler-Zebeli et al., 2021). Indeed, a recent study reported that luminal concentrations of all detected LCFAs were higher in the cecum and colon compared to the ileum and jejunum in the porcine intestines (Metzler-Zebeli et al., 2021). However, it remains unclear how LCFAs or their bioavailability vary throughout the gut in humans or in small laboratory animals commonly used for models of enteric infection. Nonetheless, recent molecular studies in combination with bacterial genetics and *in vivo* infection models have demonstrated the contributions of LCFAs in regulating the virulence potentials of bacterial pathogens in the gut. Finally, it should be noted that short chain fatty acids (SCFAs) – non-esterified fatty acids with fewer than six carbons – are even more abundant within the gut and can also act as signaling molecules that modulate virulence through diverse mechanisms (Cummings et al., 1987; Lawhon et al., 2002; Gantois et al., 2006; Hung et al., 2013; Zhang et al., 2020; Hockenberry et al., 2021; Hobbs et al., 2021), recently reviewed here (Mirzaei et al., 2021).

## Cytoplasmic LCFA Sensors

### FadR in Attaching/Effacing Pathogens

FadR functions as the master transcriptional regulator of fatty metabolism in many bacterial taxa (Iram and Cronan, 2005; Fujita et al., 2007; Shi et al., 2015). Exogenous LCFAs are first imported across the outer membrane *via* the FadL transporter, flipped across the inner membrane, and then esterified in the cytoplasm by the thioesterase FadD into long-chain acyl coenzyme A (CoA) thioesters (Fujita et al., 2007). In this activated form, LCFA-CoAs serve as substrates for beta-oxidation and membrane biosynthesis and can be directly sensed by FadR (Aalten et al., 2000). FadR is comprised of an N-terminal helix-turn-helix (HTH) DNA binding domain linked to a C-terminal effector binding domain that binds the CoA moiety of LCFA-CoAs (Aalten et al., 2000). FadR binds its target DNA sequence in its apo form to activate fatty acid biosynthesis genes (*fab*) and repress fatty acid degradation (*fad*) genes (Fujita et al., 2007). Upon binding LCFA-CoA, FadR affinity for DNA binding is decreased, thus alleviating repression of *fad* genes while diminishing activation of *fab* genes. Thus, FadR links the regulation of fatty acid metabolism with LCFA-CoA intracellular availability.

**Figure 1:**
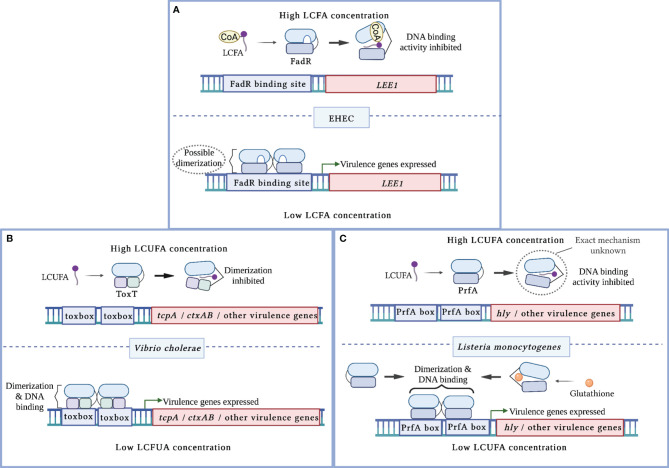
Mechanisms of virulence regulation by LCFA cytoplasmic sensors. **(A)** In enterohemorrhagic *E. coli* (EHEC), apo-FadR binds its target DNA sequences at the *LEE1* promoter region and functions as a transcriptional activator of *ler*, which encodes the master activator of the LEE pathogenicity island. In the presence of LCFA-CoAs, FadR binds the CoA moiety of LCFA-CoA. This alleviates FadR binding of DNA, resulting in decreased activation of *ler.* **(B)** In *V. cholerae*, LCUFAs bind the master virulence activator ToxT. This interaction induces a conformational change that precludes ToxT dimerization and DNA binding. As a result, ToxT-mediated activation of *tcpA, ctxAB*, and other virulence genes is decreased. **(C)** In *L. monocytogenes*, LCUFAs bind the master virulence activator PrfA. This interaction inhibits DNA binding by apo-PrfA through a mechanism that has not been fully characterized. As a result, PrfA-regulated virulence genes are not activated. Glutathione allosterically enhances PrfA binding to DNA, resulting in increased expression of PrfA-regulated virulence genes.

More recent studies have established a link between FadR and virulence in two attaching/effacing (A/E) pathogens, enterohemorrhagic *E. coli* (EHEC) and *C. rodentium* (Pifer et al., 2018; Ellermann et al., 2021). A/E pathogens utilize type 3 secretion systems (T3SS), needle-and-syringe like structures, to translocate their effector proteins directly into host cells to establish replicative niches at the colonic epithelium (Kaper et al., 2004). The genes that encode the T3SS structural components, effectors, and regulators are almost all located within the locus of enterocyte effacement (LEE) pathogenicity island, which is activated by the transcription factor Ler (Furniss and Clements, 2018). Using a screen to identify novel regulators of the LEE, Pifer et al. identified FadR as a putative transcription factor that regulates *ler* expression and demonstrated that apo-FadR binds within the *LEE1* promoter region (Pifer et al., 2018). Subsequent *in vitro* studies demonstrated that saturated and unsaturated LCFAs decrease LEE activity in a *fadR*-dependent manner (Ellermann et al., 2021). Biochemical studies further revealed that acyl-CoAs decrease FadR DNA binding at the *LEE1* promoter region, which corresponds with reduced LEE expression (Ellermann et al., 2021). Intestinal expression of the LEE is also decreased in a *C. rodentium fadR-*deficient mutant, resulting in attenuated disease (Pifer et al., 2018). Together, these findings suggest that FadR functions as an activator of *ler* (**Figure 1A**). Thus, the LEE pathogenicity island in A/E pathogens somehow became integrated into the FadR regulon, which suggests that intracellular LCFA-CoA sensing is critical for appropriate regulation of this virulence program.

The FadR regulon has also been linked to virulence through other less defined mechanisms. In some *V. cholerae* strains, genetic inactivation of *fadR* decreases ToxT-dependent virulence through undefined transcriptional and post-translational mechanisms, one of which is linked to the FadR-mediated activation of unsaturated fatty acid biosynthesis by FabA (Kovacikova et al., 2017). Loss of *fadR* also decreases virulence gene expression in *S.* Typhimurium *via* an undefined mechanism (Golubeva et al., 2016). Deletion of *fadD* attenuates virulence in EHEC, *V. cholerae*, and *S.* Typhimurium (Lucas et al., 2000; Ray et al., 2011; Gastón et al., 2013; Reens et al., 2019; Ellermann et al., 2021). Given its function as an LCFA thioesterase, the lack of functional FadD may increase intracellular non-esterified LCFAs, which also act as cytoplasmic anti-virulence signals as described below. In *V. cholerae*, decreased virulence gene expression in the *fadD* mutant is specifically associated with impaired activation of TcpP, a transcriptional inducer of *toxT*-dependent virulence (Ray et al., 2011). Finally, deletion of *fadL* in *V. cholerae, C. rodentium*, and *S.* Typhimurium attenuates pathogen growth within the mouse intestines, suggesting that import of LCFAs modulates infectivity (Reens et al., 2019; Rivera-Chávez and Mekalanos, 2019; Ellermann et al., 2020). However, loss of *fadL* in *S.* Typhimurium has also been reported to enhance its competitive advantage within the murine gut (Golubeva et al., 2016). This discrepancy may be explained by the use of different mouse and *S.* Typhmurium strains in the two studies (Golubeva et al., 2016; Reens et al., 2019). Taken together, the FadR regulon clearly contributes to the virulence potential of several enteric pathogens through mechanisms that in many cases have not been fully elucidated.

### ToxT in *V. cholerae*

*V. cholerae* initially establishes infection within the small intestines. Following epithelial attachment that is in part facilitated by its toxin co-regulated pilus (Tcp), *V. cholera* secretes cholera toxin (CT), which causes the characteristic watery diarrhea of cholera (Pierce et al., 1971; Thelin and Taylor, 1996; Kumar et al., 2020). The master virulence regulator ToxT activates CT and Tcp expression by binding toxbox sequences within the promoter regions of the *ctxAB* and *tcp* operons, respectively (Prouty et al., 2005; Withey and DiRita, 2006; Childers et al., 2011). The structure of ToxT is comprised of two HTH DNA binding motifs within the C-terminal that is connected by a linker sequence to the regulatory and dimerization domains within the N-terminal (Lowden et al., 2010; Cruite et al., 2019). ToxT dimerization is required for inducing CT and Tcp expression and for full *V. cholerae* virulence (Prouty et al., 2005; Shakhnovich et al., 2007; Childers et al., 2011).

Several *in vitro* studies initially linked long chain unsaturated fatty acids (LCUFAs) with the decreased expression of ToxT-regulated genes and consequent virulence activity (Chatterjee et al., 2007; Lowden et al., 2010; Childers et al., 2011; Plecha and Withey, 2015; Withey et al., 2015). In contrast, saturated LCFAs have been reported to either have no effect or decrease CT expression (Chatterjee et al., 2007; Lowden et al., 2010; Childers et al., 2011). Electrophoretic mobility shift assays (EMSAs) later demonstrated that LCUFAs specifically decrease the binding affinity of ToxT to DNA (Lowden et al., 2010; Plecha and Withey, 2015; Withey et al., 2015). Interestingly, the first reported crystal structure of ToxT captured the transcription factor as a monomer bound to the LCUFA *cis-*palmitoleate (Lowden et al., 2010). A hydrophobic ligand binding pocket was revealed within the N-terminal domain that interfaces the dimerization domain, where the carboxylate head of the LCUFA interacts with residues supplied by the two domains (Lowden et al., 2010). A later crystal structure captured apo-ToxT in a different conformation that favors dimerization (Cruite et al., 2019). These findings, together with supporting ToxT mutational and functional studies, revealed that LCUFAs repress virulence by inhibiting ToxT dimerization, which in turn precludes DNA binding (**Figure 1B**) (Childers et al., 2011; Cruite et al., 2019). Notably, this mechanism of action resembles that of the well-characterized ToxT inhibitor virstatin *(* Shakhnovich et al., 2007 *).* Taken together, ToxT functions as a cytoplasmic sensor that directly links intracellular LCUFAs with virulence repression in *V. cholerae.*

Recent studies have reported the rational design of potent LCUFA mimics that inhibit DNA binding by ToxT (Woodbrey et al., 2017; Woodbrey et al., 2018; Markham et al., 2021). Notably, one of these putative anti-virulence inhibitors also decreases *V. cholerae* colonization in a murine infection model (Woodbrey et al., 2018). However, the evolution of resistance remains a concern especially considering that virstatin-resistant *V. cholerae* isolates have been reported (Shakhnovich et al., 2007). Some of these isolates encode *toxT* alleles with altered dimerization domains that confer resistance to virstatin (Shakhnovich et al., 2007). Interestingly, some virstatin-resistant isolates are also impervious to the anti-virulence effects of bile (Prouty et al., 2005). However, it remains to be tested whether these isolates are specifically resistant to LCUFAs present in the bile and/or to LCUFA mimics. More broadly, these studies also raise interesting questions regarding the selective pressures that drove the emergence of virstatin-resistant ToxT alleles in the first place – and whether such pressures may also result in strain-specific responses to LCUFAs.

### HilD in *S.* Typhimurium

*S.* Typhimurium establishes infection within the small intestines and invades enterocytes by using a T3SS secretion encoded within the *Salmonella* pathogenicity island 1 (SPI-1) (Wallis and Galyov, 2000; Zhou and Galán, 2001; Yip et al., 2005; Ellermeier and Slauch, 2007). SPI-1 genes are directly activated by the transcriptional regulator HilA (Bajaj et al., 1995). The expression of *hilA* is positively regulated by interactions between the transcriptional regulators HilD, HilC, and RtsA (Ellermeier et al., 2005; Koh-Eun et al., 2020). HilD, which is structurally similar to ToxT, is considered the principal regulator that links environmental conditions to SPI-1 activity, while HilC and RtsA primarily function as signal amplifiers (Ellermeier et al., 2005; Lin et al., 2008; Golubeva et al., 2012; Koh-Eun et al., 2020; Chowdhury et al., 2021).

A recent study demonstrated that luminal LCFAs extracted from murine colonic contents potently inhibit *hilA* promoter activity (Chowdhury et al., 2021). The diffusible signal factor (DSF) *cis*-2-hexadecenic acid (c2-HDA), a C16:1 derivative, was among the most abundant molecules recovered from these colonic LCFAs (Chowdhury et al., 2021). Further studies revealed that purified c2-HDA is a strong inhibitor of *hilA* promoter activity by interfering with HilD binding of DNA (Bosire et al., 2020; Chowdhury et al., 2021). Similarly, common dietary LCFAs such as oleic acid inhibit HilD binding to DNA, thus repressing SPI-1 virulence genes (Golubeva et al., 2016). These findings correspond with the enhanced intestinal fitness of a *fadL-*deficient mutant in a *fadR* and beta-oxidation independent manner, which supports the idea that LCFA derivatives may function as anti-virulence signals in the gut *via* their interactions with HilD (Golubeva et al., 2016; Bosire et al., 2020). *In silico* modeling and HilD mutational studies identified common residues in HilD that likely interact with the carboxylic heads of LCFAs and DSFs (Chowdhury et al., 2021). Distinct residues in HilD were also identified that uniquely interact with LCFAs or with DSFs (Chowdhury et al., 2021). Notably, unlike dietary LCFAs with a *cis*-2 saturation, c2-HDA also promotes the proteolytic degradation of HilD and decreases the DNA binding activities of the other two SPI-1 regulators RtsA and HilC (Golubeva et al., 2016; Bosire et al., 2020; Chowdhury et al., 2021; Chowdhury et al., 2021). Finally, c2-HDA also inhibits the promoter activity of the ToxT-regulated *ctxAB* operon, suggesting that this DSF may also repress virulence in *V. cholerae* by targeting ToxT activity (Bosire et al., 2020).

DSFs like c2-HDA are produced by several Gammaproteobacteria including opportunistic respiratory pathogens such as *Pseudomonas aeruginosa* and *Burkholderia cenocepacia* (Boon et al., 2008; Twomey et al., 2012). While DSFs have been detected in the murine gut, their producers remain to be identified (Chowdhury et al., 2021). Given their anti-virulence effects, DSFs may represent a mechanism of colonization resistance that is imparted by some members of the gut microbiome against closely related pathogens. It is also possible that *Salmonella* and *V. cholerae* utilize DSFs as signals that inform them of their luminal localization, where the maximal expression of virulence factors that facilitate epithelial colonization is suboptimal. Thus, it will be interesting to determine whether DSFs also modulate the virulence of other enteric pathogens. More broadly, given that different classes of LCFAs inhibit the DNA binding activities of two AraC-like virulence activators – HilD and ToxT – it will be interesting to determine whether LCFAs also inhibit similar virulence regulators in other pathogens to impart their anti-virulence effects.

### PrfA in *L. monocytogenes*

*L. monocytogenes* initiates infection in the small intestines by first invading epithelial cells, and then escaping into the cytoplasm to replicate and disseminate into adjacent cells *via* actin-based motility (Dussurget et al., 2004). PrfA is the master virulence regulator that activates genes required for intracellular replication (Scortti et al., 2007). PrfA is comprised of a C-terminal domain that contains the HTH DNA-binding motif and an N-terminal domain that contains the dimerization domain and a ligand binding pocket (Scortti et al., 2007; Tran et al., 2022). PrfA binds its target DNA sequences called PrfA boxes as a homodimer in a hierarchical fashion (Sheehan et al., 1995). Early virulence genes including *hly* that are required for pathogen escape into the cytoplasm contain PrfA boxes with high affinity for apo-PrfA within their promoters (Scortti et al., 2007). Virulence genes that are activated later in the intracellular replication cycle contain low affinity PrfA boxes within their promoters (Scortti et al., 2007). Thus, PrfA requires allosteric co-activation by glutathione to bind these low affinity DNA sequences (Reniere et al., 2015; Hall et al., 2016).

Although LCFAs can exert bactericidal effects against *L. monocytogenes*, some LCFAs also function as anti-virulence signals at subinhibitory concentrations (Lillebæk et al., 2017; Santos et al., 2020). Similar to ToxT in *V. cholerae*, PrfA-regulated genes are repressed following treatment with different unsaturated, but not saturated, LCFAs (Lillebæk et al., 2017; Santos et al., 2020; Chen et al., 2021). A follow-up study demonstrated that LCUFAs inhibit DNA binding at the *hly* promoter region by a PrfA allele with enhanced DNA affinity (Santos et al., 2020). The precise mechanism by which LCUFAs interact with PrfA to modulate its DNA binding activity remains to be elucidated (**Figure 1C**). Notably, small molecule inhibitors have been identified that induce a conformational shift that precludes PrfA binding to DNA (Good et al., 2016; Kuleín et al., 2018; Tran et al., 2022). Thus, LCUFAs may also act through a similar mechanism. Collectively, these findings demonstrate that LCFAs attenuate virulence in Gram-positive organisms, in a similar manner to Gram-negative enteric pathogens, by allosterically inhibiting the activities of pro-virulence transcriptional activators.

## Extracellular Sensing of LCFAs by Histidine Kinases

Two-component systems (TCS), typically comprised of a histidine kinase (HK) and a cytoplasmic response regulator (RR), are mechanisms by which extracellular signals are coupled to gene regulation in bacteria (Wolanin et al., 2002). HKs are transmembrane receptors that become activated *via* the autophosphorylation of a histidine residue in response to specific ligands. The signal is then transduced *via* the transfer of the phosphate from the HK to the RR, which in turn modulates the DNA binding affinity of the RR to regulate gene expression.

### 2-Arachidonoyl Glycerol Sensing by QseC in A/E Pathogens

**Figure 2:**
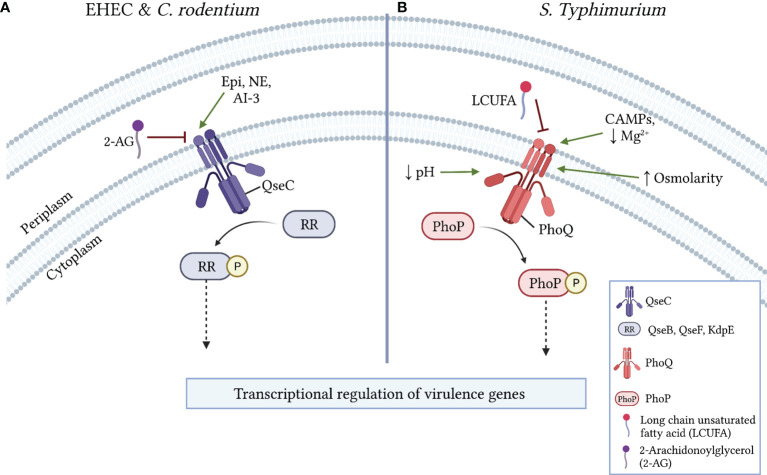
Mechanisms of virulence regulation *via* LCFA sensing by histidine kinase receptors. **(A)** In EHEC and *C. rodentium*, the activation of the pro-virulence histidine kinase receptor QseC is either induced by epinephrine (Epi), norepinephrine (NE), or autoinducer 3 (AI-3) or inhibited by 2-arachidonylglycerol (2-AG). Following its activation, QseC phosphorylates the response regulators (RR) QseB, QseF, and/or KdpE, resulting in the downstream transcriptional regulation of virulence genes. **(B)** In *S.* Typhimurium, the histidine kinase receptor PhoQ is inhibited by LCUFAs or activated in the periplasm by low Mg^2+^, other divalent cations, or the presence of cationic antimicrobial peptides (CAMPs). PhoQ is also activated by low cytoplasmic pH (~5) or by osmotic stress. Following its activation, PhoQ phosphorylates the RR PhoP, which results in the downstream transcriptional regulation of virulence genes.

QseC is a HK receptor that autophosphorylates following interactions with the host neurotransmitters epinephrine and norepinephrine or with the quorum sensing signal autoinducer-3 (Clarke et al., 2006; Hughes et al., 2009). In EHEC and *C. rodentium*, QseC activation stimulates complex intracellular signaling cascades that ultimately induce *ler* expression to activate the LEE (Hughes et al., 2009; Moreira et al., 2016). A recent study identified the endocannabinoid 2-AG, an arachidonic acid derivative, as a host-derived signal that attenuates virulence in several enteric pathogens (Ellermann et al., 2020). In EHEC and *C. rodentium*, 2-AG inhibits the activation of LEE-encoded genes and decreases T3SS activity in a *qseC*-dependent manner (Ellermann et al., 2020). Autophosphorylation assays further revealed that 2-AG specifically inhibits QseC activation to impart its anti-virulence effects (**Figure 2A**). Supporting these findings, mice with elevated 2-AG levels in the colon were resistant to *C. rodentium* infection, a protective effect that was lost in mice challenged with a *qseC-*deficient mutant (Ellermann et al., 2020). 2-AG also attenuates SPI-1 and SPI-2 associated functions in *S.* Typhimurium through an undefined mechanism (Ellermann et al., 2020). Notably, virulence regulation in *S.* Typhimurium has also been linked to QseC activity (Moreira et al., 2010; Moreira and Sperandio, 2012). Given the identification of small molecule inhibitors of QseC that attenuate virulence in various pathogens (Rasko et al., 2008; Curtis et al., 2014), it will be interesting to determine whether 2-AG and other host-derived arachidonic acid derivatives act as general anti-virulence signals in pathogens in which QseC activity modulates virulence.

### LCUFA Sensing by PhoQ in *S.* Typhimurium

PhoPQ is a TCS that regulates a complex network of genes involved in virulence, cationic antimicrobial peptide (CAMP) resistance, magnesium homeostasis, and outer membrane physiology (Groisman et al., 2021). Accordingly, the HK receptor PhoQ becomes autophosphorylated in response to various environmental signals including moderate acid stress, osmotic stress, low Mg^2+^ availability, and CAMPs (Fields et al., 1986; Fields et al., 1989). More recently, a screen of potential plant-derived inhibitors of PhoQ identified LCUFAs, and not saturated LCFAs, as exogenous signals that inhibit autokinase activity in PhoQ and repress PhoP regulated genes (Gastón et al., 2013; Carabajal et al., 2020). Interestingly, this included conjugated linoleic acids (CLAs), which are common products of bacterial biotransformation reactions that occur in the gut. However, the administration of a CLA supplement failed to attenuate *S.* Typhimurium gut infection or systemic dissemination in the streptomycin infection model (Carabajal et al., 2020). Subsequent studies demonstrated that the periplasmic sensor domain in PhoQ likely interacts with various LCUFAs to inhibit its autophosphorylation (Carabajal et al., 2020). (**Figure 2B**) Finally, in addition to its phosphorylation by PhoQ, recent work has demonstrated that PhoP DNA binding activity is further modulated by the acetylation of lysine residues within its DNA binding domain (Ren et al., 2016; Li et al., 2021). PhoP acetylation diminishes its DNA binding activity, resulting in attenuated virulence in *S.* Typhimurium. One mechanism of PhoP acetylation involves the enzymatic transfer of an acetyl moiety from an acetyl-CoA donor (Ren et al., 2016). Notably, acetyl-CoA is a product of LCFA beta-oxidation, thus introducing a further putative mechanism by which LCFA metabolism may modulate bacterial virulence. Taken together, as with QseC, the PhoPQ TCS is expressed in various Gram-negative enteric bacteria and is stimulated by many of the same activating signals (Groisman et al., 2021). Therefore, it will be interesting to investigate whether LCUFAs and post-translational modifications such as acetylation also inhibit the PhoPQ TCS in diverse enteric bacteria, and if so, to delineate the resulting transcriptional and functional effects of such interactions.

## Conclusions

Collectively, these molecular studies clearly demonstrate that LCFAs often act as anti-virulence signals that inhibit key transcriptional activators of virulence and histidine kinases that stimulate pro-virulence intracellular cascades. Strikingly, the anti-virulence effects of LCUFAs in particular are shared by many enteric pathogens with distinct physiologies, virulence programs, infectious processes, and lifestyles. However, many fundamental questions remain unanswered including: Why have many gut pathogens evolved to respond to LCFAs as anti-virulence signals? And how do different host-, microbe- and diet-derived LCFA molecules precisely modulate virulence during the pathogen life cycle in the gut? The latter question is especially difficult to answer because it remains poorly understood how LCFA profiles vary spatially throughout the gut and how these profiles are altered during infection. Moreover, it remains unknown to what extent intestinal LCFAs are bioavailable for uptake by bacterial pathogens to function as anti-virulence signals – or as nutrients or antimicrobials. Nonetheless, *in vivo* infection studies that have utilized LCFA “blind” mutants or that have increased the content of specific LCFA-derivatives have shown that LCFAs have the potential to attenuate the virulence of enteric pathogens during gut infection. Thus, a more complete understanding of how pathogens and commensals sense and respond to LCFAs in the gut may enable the design of new anti-virulence approaches that target intestinal pathogens while minimally impacting the endogenous gut microbiota.

## Author Contributions

MM prepared the figures and helped write the manuscript. ME wrote and edited the manuscript. All authors contributed to the article and approved the submitted version.

## Funding

This work was supported by a target faculty grant awarded to M.E. from an NIH COBRE grant (5P20GM103641-09; awarded to Drs. Mitzi and Prakash Nagarkatti).

## Author Disclaimer

The funders had no role in the preparation of the manuscript.

## Conflict of Interest

The authors declare that the research was conducted in the absence of any commercial or financial relationships that could be construed as a potential conflict of interest.

## Publisher’s Note

All claims expressed in this article are solely those of the authors and do not necessarily represent those of their affiliated organizations, or those of the publisher, the editors and the reviewers. Any product that may be evaluated in this article, or claim that may be made by its manufacturer, is not guaranteed or endorsed by the publisher.
